# Characterization of Sub-Monolayer Contaminants at the Regrowth Interface in GaN Nanowires Grown by Selective-Area Molecular Beam Epitaxy

**DOI:** 10.3390/cryst8040178

**Published:** 2018

**Authors:** Paul Blanchard, Matt Brubaker, Todd Harvey, Alexana Roshko, Norman Sanford, Joel Weber, Kris A. Bertness

**Affiliations:** National Institute of Standards and Technology (NIST), Boulder, CO 80305, USA

**Keywords:** gallium nitride, defects, nanowires, atom probe tomography

## Abstract

While GaN nanowires (NWs) offer an attractive architecture for a variety of nanoscale optical, electronic, and mechanical devices, defects such as crystal polarity inversion domains (IDs) can limit device performance. Moreover, the formation of such defects during NW growth is not fully understood. In this study, we use transmission electron microscopy (TEM) and atom probe tomography (APT) to investigate the effects of sub-monolayer contamination at the regrowth interface in GaN NWs grown by selective-area molecular beam epitaxy (MBE). TEM energy dispersive X-ray spectroscopy (EDS) and APT independently identified Al and O contamination localized at the regrowth interface in two of the three growth runs examined. The Al and O concentrations were each estimated to be on the order of 11% of an ideal *c*-plane monolayer in the most severely contaminated case. The amount of contamination correlated with the number of crystal polarity inversion domain defects (IDs) across the growth runs. A growth run in which the pre-regrowth HF vapor etch step was replaced by HCl immersion showed the smallest quantity of O and no measurable Al. In addition, many of the NWs examined from the HCl-treated growth run turned out to be free of IDs. These results suggest that sub-monolayer contamination introduced during processing contributes to defect formation in MBE-grown GaN NWs.

## 1. Introduction

Recent advancements in selective-area growth of GaN nanowires (NWs) grown by molecular beam epitaxy (MBE) have yielded unprecedented control over NW morphology and placement [1–6], enabling new and improved device applications [7–10]. However, because selective-area growth requires ex-situ processing of the substrate in order to pattern features in the growth mask, there is a risk of introducing contamination at the substrate-NW regrowth interface. In this study, we investigate such contamination and its possible effects on the formation of crystal polarity inversion domains (IDs) in GaN NWs. When present, IDs in GaN may induce unintentional defect-related doping, reduce the free carrier mobility, and change the incorporation of alloy materials such as indium during growth [11], all of which can inhibit the performance of electronic and optoelectronic devices.

## 2. Materials and Methods

The GaN NWs studied here were grown by N-polar selective-area MBE [1]. Starting with a Si (111) substrate, a GaN thin-film buffer layer was grown with the following approximate structure: (1) 50 nm AlN; (2) 100 nm GaN; (3) 5× alternating AlGaN/GaN layers of 5 nm thickness each; (4) 150 nm GaN. After buffer layer growth, the substrate was removed from the MBE system, and an approximately 70 nm-thick SiN mask was deposited by PECVD on the GaN buffer surface. Arrays of holes in the SiN mask were created via electron-beam lithography and reactive ion etching (RIE), followed by a solvent rinse and an O_2_ RIE plasma treatment to remove organic residue. Next, the substrate was subjected to an acid etch consisting of either exposure to HF vapor or immersion in HCl:H_2_O (1:1) before returning to the MBE growth chamber. Finally, GaN nanowires were grown from the holes in the SiN mask. Among the NWs that were characterized here, lengths were on the order of 5 μm to 6 μm, with diameters between about 150 nm and 250 nm. Three separate NW growth runs, referred to as samples S1, S2, and S3, were considered in this experiment.

In order to evaluate the crystalline quality of the selectively-grown nanowire arrays, cross-sectional transmission electron microscope (TEM) lamellae were prepared directly from the as-grown substrates by focused ion beam (FIB) lift-out techniques [12]. Each lamella was positioned such that it cut through several neighboring NWs, resulting in 4 or 5 axially cross-sectioned NWs per lamella, each with an intact regrowth interface.

To further characterize the apparent contamination at the NW/ buffer regrowth interface, laser-assisted atom probe tomography (L-APT) was performed on samples of material containing the NW regrowth interface from growth runs S1, S2, and S3. In order to preserve the intact interface in each sample, a small piece of the growth substrate containing a single NW was extracted and transferred to a pre-fabricated silicon micropost using a procedure similar to the typical FIB lift-out technique [13]. Annular FIB milling with simultaneous SEM imaging was then used to carefully shave away material until the remaining cone-shaped sample had an apex diameter on the order of 40 nm, with the NW regrowth interface located within a few hundred nm of the apex, as shown in Figure 2a. L-APT measurements were carried out on a straight-flight-path system with 355 nm pulsed laser illumination. The operating conditions were as follows: flight length = 90 mm; laser pulse energy = 300 fJ; sample temperature ≈ 54 K; laser pulse repetition rate = 250 kHz; target detection rate = 0.5%. Previous work has shown that L-APT operating conditions similar to these yield approximately the correct aluminum mole fraction in bulk thin-film Al_*x*_Ga_1−*x*_N samples [14].

## 3. Results

### 3.1. TEM Imaging

**Figure 1.**
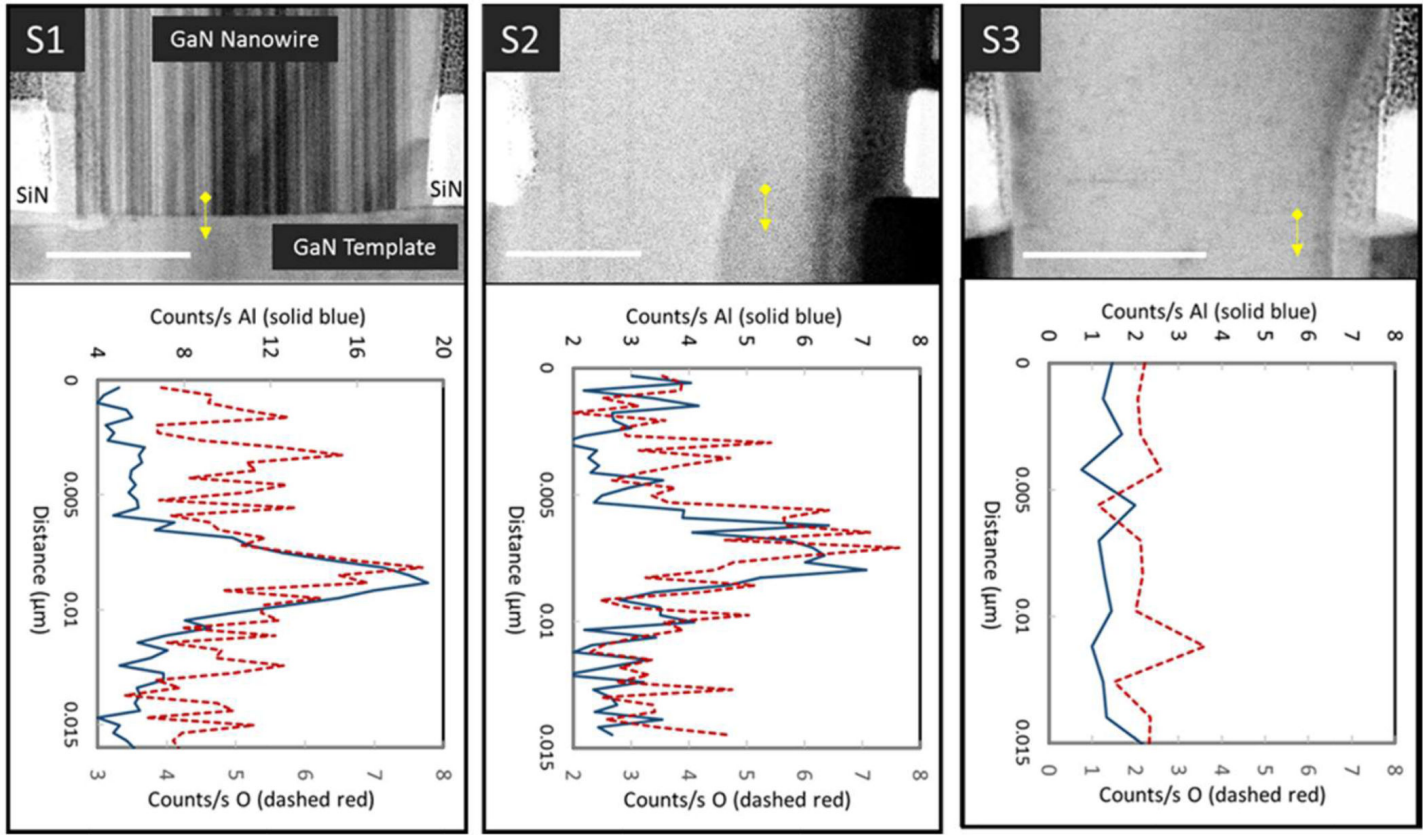
Typical bright-field STEM images and EDS line scan data from the regrowth interface of nanowires in growth runs S1, S2, and S3. The scale bars are 100 nm. The vertical lines running through the NWs are polarity inversion domains, as verified by direct lattice imaging. The arrow overlaying each STEM image depicts an example EDS line scan location. Data from one such EDS scan are plotted below each image; additional scans with similar results were taken at multiple positions across each NW.

Direct lattice imaging via annular bright field scanning TEM (ABF STEM) on a JEM-ARM200F system (JEOL, Peabody, MA, USA; reference to a commercial product is included for informational purposes only, and does not constitute endorsement by NIST) [15] confirmed that the primary growth direction of the NWs was along the N-polar wurtzite *c*-axis. ABF imaging also revealed polarity inversion domain defects within some of the NWs, as shown in Figure 1. These Ga-polar inversion domains (IDs) typically appear to nucleate at the regrowth interface and propagate along the entire length of the NW *c*-axis. Growth run S1 showed by far the largest number of IDs, with dozens visible in each cross-sectioned NW. S2 had fewer IDs, with between two and four visible per NW. S3 showed the fewest IDs; of the four S3 NWs inspected, two showed no visible IDs, and the other two showed one ID each.

To look for possible contamination at the NW/buffer regrowth interface in these growth runs, STEM energy dispersive X-ray spectroscopy (EDS) was performed. Here, the electron beam was scanned in a vertical line across the regrowth interface to create a relative concentration profile, as shown in Figure 1. Several EDS scans were performed in different positions across each NW cross-section, with similar results. The average results from each growth run are summarized in Table 1. Growth run S1 showed EDS signals associated with Al and O, each reaching its maximum at the regrowth interface. S2 also showed evidence of interfacial Al and O, but at lower levels than S1. S3 showed no EDS signals above the background for either Al or O. There was no apparent difference between the Al and O levels in EDS scans performed at the root of IDs and those performed in non-ID interface regions.

In addition to EDS scans of the NW/ buffer regrowth interface, similar measurements were performed across the interface between the SiN mask and the GaN thin film buffer layer in growth runs S1 and S2. Interestingly, each of these measurements showed a substantial EDS signal from O, but did not show measurable Al under the SiN mask to within the EDS chemical sensitivity (which is on the order of 1 to 2 atomic %). Note that the EDS measurements performed here are not referenced to a composition standard and should be treated as qualitative.

### 3.2. Atom Probe Tomography Characterization

**Figure 2.**
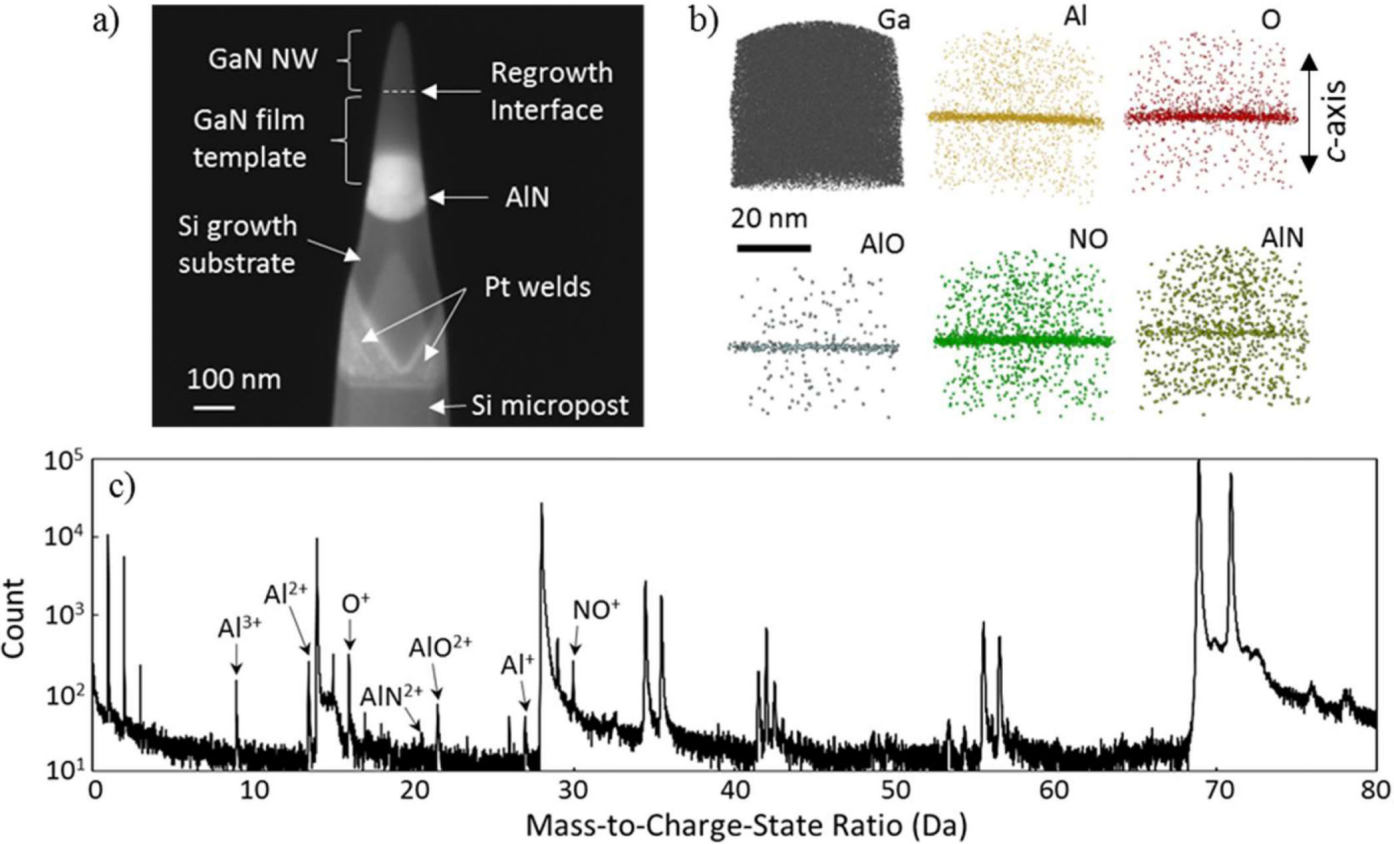
(**a**) FESEM image of L-APT nanowire regrowth interface sample from growth run S1 after FIB lift-out and annular milling; (**b**) Reconstructed positions of L-APT detector hits from mass spectrum peaks hypothesized to be due to Al, O, and related molecular species in the S1 sample. The reconstruction is limited to a region of interest (ROI) near the regrowth interface. The reconstructed positions of likely Ga atoms are also shown for comparison; (**c**) Accumulated mass spectrum histogram from the ROI (including background signal), showing the peaks hypothesized to be related to Al and O.

The L-APT reconstruction from a region of interest (ROI) around the regrowth interface in a S1 NW is shown in Figure 2b. As is typical in a GaN L-APT spectrum [16], peaks due to H, Ga, N, and related molecules are present. In addition, mass spectrum peaks associated with Al in the 1+, 2+, and 3+ charge states are present, as well as a peak at 16 Da that is likely due to O^+^. Peaks consistent with AlN^+^, AlO^2+^, and NO^2+^ were also identified, as shown in Figure 2c. It is worth noting that there is no evidence of carbon or hydrocarbon compounds. Along the *c*-axis, the likely Al and O-related species are highly localized near the regrowth interface. The radial distributions, by contrast, appear to be random and uniform, with no evidence of significant clustering in the {0001} plane.

In order to estimate the magnitude of the contamination, it is useful to express the approximate quantities of Al and O in each sample as fractions of an ideal *c*-plane monolayer. It should be emphasized that calculating the ideal monolayer fraction neither implies nor requires that the regrowth interface is actually a single pristine *c*-plane. In fact, there is a spread of approximately 2 nm in the reconstructed axial positions of Al, O, and related species near the interface, which may be due to non-zero roughness of the GaN buffer surface, to diffusion of Al and O during nanowire growth, or simply to artifacts introduced by the atom probe reconstruction process. Because it ignores these ambiguities in the actual spatial distributions of the contaminants, the ideal monolayer fraction $f_{ML}$ is a useful metric for comparing contamination levels across samples. It is estimated by

(1) $$f_{ML}\approx \frac{N_{APT}a^{2}\cos\left(30^{\circ }\right)}{\eta_{APT}\pi r^{2}n},$$

where $N_{APT}$ is the total number of Al or O atoms detected in the reconstructed interface region (including those that are part of molecular species), $\eta_{APT}$ is the approximate ion detection efficiency in L-APT (assumed here to be 50%), a is the wurtzite GaN a lattice parameter (~0.319 nm), r is the radius of the cone-shaped L-APT specimen at the regrowth interface (measured by SEM), and n is the number of Ga (or N, depending on the terminating layer) *c*-plane surface atoms in one primitive unit cell (*n* = 1). Here, the “reconstructed interface region” was defined to be within ± 5 nm along the *c*-axis from the approximate center of the visible interface layer. All atomic counts were background-corrected by use of the default local range-assisted model in the IVAS^™^ reconstruction software, version 3.6.14 (Cameca, Madison, WI, USA; reference to a commercial product is included for informational purposes only, and does not constitute endorsement by NIST).

The estimated ideal monolayer fractions for Al and O are summarized in Table 1, and are consistent with the STEM EDS data. The NW regrowth interface in growth run S1 showed significantly more Al and O than S2. S3 showed no evidence of Al, and showed substantially reduced O compared to the other two growth runs. In addition to the nanowire regrowth interface measurements, an additional set of L-APT measurements was collected from the interface between the SiN mask and the GaN thin film buffer from growth run S1. L-APT showed significant O at the SiN/GaN interface, but no evidence of Al contamination.

It should be emphasized that the uncertainty in these L-APT $f_{ML}$ estimates has not been quantified, but is undoubtedly large. One significant source of uncertainty is the ambiguous identification of L-APT mass spectrum peaks. For example, some or all of the ions attributed to O^+^ at 16 Da could alternatively be attributed to NH_2_^+^. O^+^ was chosen as the most likely identity of ions detected at 16 Da because of their highly localized location near the regrowth interface, and because TEM EDS independently detected oxygen at the regrowth interfaces in all cases except for S3. However, it is possible that the oxygen $f_{ML}$ has been substantially overestimated due to contributions from complexes such as NH_2_^+^; in the limiting case where all of the O^+^ is reassigned to NH_2_^+^ and all of the NO^+^ is reassigned to N_2_H_2_^+^, the oxygen $f_{ML}$ becomes 0.01 in S1, 0.004 in S2, and 0.0 in both S3 and S1-m (the SiN mask region). Other sources of error, such as detector dead time effects [17], may also influence the relative compositional measurements. As such, the L-APT $f_{ML}$ estimates should be viewed as somewhat qualitative.

## 4. Discussion

There are several important caveats to keep in mind before drawing any firm conclusions from these results about the relationship between regrowth interface contamination and the formation of IDs. First of all, the sample size is small, due to the time-intensive nature of L-APT and TEM EDS. Secondly, the uncertainty in each of these L-APT and TEM EDS composition measurements has not been quantified. Finally, there are many different factors besides contamination that can influence inversion domain formation [18–20].

Nevertheless, the data summarized in Table 1 show an apparent correlation between the number of inversion domains and the amount of Al and/or O contamination at the regrowth interface. This suggests a causal relationship; indeed, Al_2_O_3_ has been used in the past to intentionally switch the polarity of GaN [21]. Here, the Al and O contamination constitutes much less than a complete monolayer, suggesting that even a relatively minor quantity of interface contamination can influence the formation of IDs. Al and O were the only contaminants observed in this study, so it is not clear whether ID formation is particular to these species, or whether sub-monolayer contamination by species such as Si or C would have a similar effect.

Achieving a pristine regrowth interface is challenging in a selective-area-growth process due to the numerous opportunities for contamination during ex-situ substrate processing. In our case, oxygen contamination may be due to air exposure and/or to the O_2_ RIE plasma cleaning step. The source of the Al contamination is less obvious. However, the fact that Al is not present at the SiN/GaN interface in the S1 and S2 mask regions narrows down the possibilities, because it shows that the Al contamination must have occurred after the SiN mask was deposited and etched. As such, we can rule out Al contamination during the GaN buffer growth process. One possible culprit is the O_2_ RIE plasma cleaning step; the RIE chamber has an aluminum electrode, and it is possible that a small amount of unintentional sputtering of Al onto the substrate occurred.

It is curious that S1 and S2 show substantially different levels of contamination, despite the fact that the growth runs were subjected to nominally identical ex-situ processing steps. This indicates that some subtle variation in processing conditions was sufficient to substantially impact the relative contamination level. Uncontrolled factors, such as the aggregate effect of processes performed by other users of the O_2_ RIE system over time or the precise placement of the wafer in the RIE chamber, may play a role in determining the amount of contamination. Hence, while the absence of Al and the reduced O in S3 suggest that an HCl-based treatment may be effective at removing such contamination prior to regrowth, we cannot rule out the possibility that the reduction in contamination in S3 is due to an unknown fluctuation in a prior processing step.

## 5. Conclusions

In summary, we have used L-APT and TEM to characterize the NW regrowth interface in NWs grown by selective area MBE. TEM imaging revealed inversion domain defects in the NWs, the number of which varied substantially between growth runs. Both STEM EDS and L-APT independently identified Al and O contamination localized at the regrowth interface in two of the three growth runs examined, with no evidence of C or other contaminants. The Al and O concentrations were each estimated to be on the order of 11% of an ideal *c*-plane monolayer in the most severely contaminated growth run, which was also the growth run with the highest density of inversion domain defects. The growth run in which the pre-regrowth HF vapor etch was replaced by HCl showed no measurable Al, and the lowest level of O contamination. In addition, some of the NWs in the HCl-treated growth run turned out to be free of inversion domain defects. These results are consistent with the hypothesis that sub-monolayer Al and O contamination at the regrowth interface contribute to the formation of inversion domain defects.

## Figures and Tables

**Table 1.** Summary of TEM and L-APT measurements of the regrowth interface in three different selective-area nanowire growth runs, plus the SiN mask region from sample S1. Each acid treatment was applied prior to NW growth. The TEM EDS counts per second above background represent an average of the maximum background-corrected counts in several EDS line scans of 4 or 5 NWs (or multiple locations in the SiN mask region). The L-APT ideal *c*-plane monolayer fraction ($f_{ML}$) results are from one regrowth interface sample each.

Sample Description	Acid Treatment	Approximate # of Visible Inversion Domains per NW Cross Section	TEM EDS Counts per Second		Estimated $f_{ML}$ by L-APT	

			Al	O	Al	O

S1: NW/GaN buffer interface	HF vapor	25+	11	6	0.11	0.12

S2: NW/GaN buffer interface	HF vapor	2 to 4	3	3	0.05	0.09

S3: NW/GaN buffer interface	HCl dip	0 to 1	0	0	0	0.05

S1-m: SiN mask/GaN buffer interface	-	-	0	5	0	0.14

## References

[1] Brubaker MD; Duff SM; Harvey TE; Blanchard PT; Roshko A; Sanders AW; Sanford NA; Bertness KA Polarity-Controlled GaN/AlN Nucleation Layers for Selective-Area Growth of GaN Nanowire Arrays on Si(111) Substrates by Molecular Beam Epitaxy. Cryst. Growth Des 2016, 16, 596–604.

[2] Albert S; Bengoechea-Encabo A; Sanchez-Garcia MA; Kong X; Trampert A; Calleja E Selective area growth of In(Ga)N/GaN nanocolumns by molecular beam epitaxy on GaN-buffered Si(111): From ultraviolet to infrared emission. Nanotechnology 2013, 24, 175303. 10.1088/0957-4484/24/17/175303 23558410

[3] Bengoechea-Encabo A; Albert S; Sanchez-Garcia MA; Lopez LL; Estrade S; Rebled JM; Peiro F; Nataf G; de Merry P; Zuniga-Perez J; Selective area growth of a- and c-plane GaN nanocolumns by molecular beam epitaxy using colloidal nanolithography. J. Cryst. Growth 2012, 353, 1–4.

[4] Bertness KA; Sanders AW; Rourke DM; Harvey TE; Roshko A; Schlager JB; Sanford NA Controlled Nucleation of GaN Nanowires Grown with Molecular Beam Epitaxy. Adv. Funct. Mater 2010, 20, 2911–2915.

[5] Kishino K; Sekiguchia H; Kikuchi A Improved Ti-mask selective-area growth (SAG) by rf-plasma-assisted molecular beam epitaxy demonstrating extremely uniform GaN nanocolumn arrays. J. Cryst. Growth 2009, 311, 2063–2068.

[6] Wu CH; Lee PY; Chen KY; Tseng YT; Wang YL; Cheng KY Selective area growth of high-density GaN nanowire arrays on Si(111) using thin AlN seeding layers. J. Cryst. Growth 2016, 454, 71–81.

[7] Kishino K; Yanagihara A; Ikeda K; Yamano K Monolithic integration of four-colour InGaN-based nanocolumn LEDs. Electron. Lett 2015, 51, 852–854.

[8] Kum H; Seong HK; Lim W; Chun D; Kim YI; Park Y; Yoo G Wafer-scale Thermodynamically Stable GaN Nanorods via TwoStep Self-Limiting Epitaxy for Optoelectronic Applications. Sci. Rep 2017, 7, 40893. 28098259 10.1038/srep40893 PMC5241666

[9] Zhao SR; Nguyen HPT; Kibria MG; Mi ZT III-Nitride nanowire optoelectronics. Prog. Quantum Electron 2015, 44, 14–68.

[10] Hauswald C; Giuntoni I; Flissikowski T; Gotschke T; Calarco R; Grahn HT; Geelhaar L; Brandt O Luminous Efficiency of Ordered Arrays of GaN Nanowires with Subwavelength Diameters. ACS Photonics 2017, 4, 52–62.

[11] Kong X; Li H; Albert S; Bengoechea-Encabo A; Sanchez-Garcia MA; Calleja E; Draxl C; Trampert A Titanium induced polarity inversion in ordered (In,Ga)N/GaN nanocolumns. Nanotechnology 2016, 27, 7. 10.1088/0957-4484/27/6/065705 26759358

[12] Giannuzzi LA; Drown JL; Brown SR; Irwin RB; Stevie F Applications of the FIB lift-out technique for TEM specimen preparation. Microsc. Res. Tech 1998, 41, 285–290. 9633946 10.1002/(SICI)1097-0029(19980515)41:4<285::AID-JEMT1>3.0.CO;2-Q

[13] Thompson K; Lawrence D; Larson DJ; Olson JD; Kelly TF; Gorman B In situ site-specific specimen preparation for atom probe tomography. Ultramicroscopy 2007, 107, 131–139. 16938398 10.1016/j.ultramic.2006.06.008

[14] Sanford NA; Blanchard PT; Davydov AV Laser-Assisted Atom Probe Tomography of AlN and AlGaN. In Proceedings of the 59th Electronic Materials Conference, University of Notre Dame, South Bend, IN, USA, 28–30 June 2017.

[15] Roshko A; Brubaker MD; Blanchard PT; Bertness KA; Harvey TE; Geiss RH; Levin I Comparison of convergent beam electron diffraction and annular bright field atomic imaging for GaN polarity determination. J. Mater. Res 2017, 32, 936–946. 10.1557/jmr.2016.443 PMC6604648 31274956

[16] Diercks DR; Gorman BP; Kirchhofer R; Sanford N; Bertness K; Brubaker M Atom probe tomography evaporation behavior of *C*-axis GaN nanowires: Crystallographic, stoichiometric, and detection efficiency aspects. J. Appl. Phys 2013, 114, 184903-1-9.

[17] Meisenkothen F; Steel EB; Prosa TJ; Henry KT; Kolli RP Effects of detector dead-time on quantitative analyses involving boron and multi-hit detection events in atom probe tomography. Ultramicroscopy 2015, 159, 101–111. 26342554 10.1016/j.ultramic.2015.07.009

[18] Matsubara T; Denpo Y; Okada N; Tadatomo K V-shaped pits in HVPE-grown GaN associated with columnar inversion domains originating from foreign particles of α-Si_3_N_4_ and graphitic carbon. Micron 2017, 94, 9–14. 27974253 10.1016/j.micron.2016.11.008

[19] Sanchez AT; Dimitrakopoulos GP; Ruterana P Mechanisms for the formation of inversion domains in GaN. In Microscopy of Semiconducting Materials 2003; Cullis AG., Midgley PA, Eds.; CRC Press: Boca Raton, FL, USA, 2003; pp. 269–272.

[20] Zhou H; Phillipp F; Schroder H; Bell JM Influence of domain boundaries on polarity of GaN grown on sapphire. Appl. Surf. Sci 2005, 252, 483–487.

[21] Hite JK; Garces NY; Goswami R; Mastro MA; Kub FJ; Eddy CR Selective switching of GaN polarity on Ga-polar GaN using atomic layer deposited Al_2_O_3_. Appl. Phys. Express 2014, 7, 025502-1-4.

